# Allyl isothiocyanate affects the cell cycle of *Arabidopsis thaliana*

**DOI:** 10.3389/fpls.2015.00364

**Published:** 2015-05-19

**Authors:** Signe E. Åsberg, Atle M. Bones, Anders Øverby

**Affiliations:** Department of Biology, Norwegian University of Science and TechnologyTrondheim, Norway

**Keywords:** *Arabidopsis thaliana*, isothiocyanates, cell cycle, plant defense, Allyl isothiocyanate

## Abstract

Isothiocyanates (ITCs) are degradation products of glucosinolates present in members of the Brassicaceae family acting as herbivore repellents and antimicrobial compounds. Recent results indicate that allyl ITC (AITC) has a role in defense responses such as glutathione depletion, ROS generation and stomatal closure. In this study we show that exposure to non-lethal concentrations of AITC causes a shift in the cell cycle distribution of *Arabidopsis thaliana* leading to accumulation of cells in S-phases and a reduced number of cells in non-replicating phases. Furthermore, transcriptional analysis revealed an AITC-induced up-regulation of the gene encoding cyclin-dependent kinase A while several genes encoding mitotic proteins were down-regulated, suggesting an inhibition of mitotic processes. Interestingly, visualization of DNA synthesis indicated that exposure to AITC reduced the rate of DNA replication. Taken together, these results indicate that non-lethal concentrations of AITC induce cells of *A. thaliana* to enter the cell cycle and accumulate in S-phases, presumably as a part of a defensive response. Thus, this study suggests that AITC has several roles in plant defense and add evidence to the growing data supporting a multifunctional role of glucosinolates and their degradation products in plants.

## Introduction

**Figure 1:**
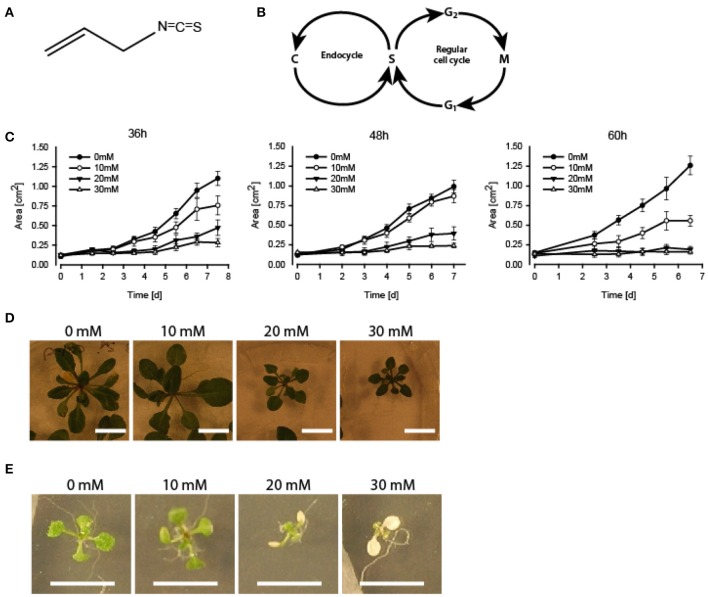
**Growth inhibition and bleaching of ***A. thaliana*** seedlings subjected to AITC**. **(A)** Chemical structure of AITC. **(B)** Outline of the plant cell cycle including the endocycle where DNA is copied (“C” in the figure) without the cell proceeding to mitosis. **(C–E)** 7 day-old seedlings subjected to vapor of 0, 10, 20, or 30 mM AITC for 36, 48, and 60 h and followed by recovery in an AITC free atmosphere for up to 11 days. **(C)** Seedling size was measured once a day for 6–7 days. **(D)** Dose-dependent growth-inhibited plants were observed following 48 h treatments and 11 d of recovery. **(E)** Bleaching of cotyledons after 48 h 20 mM AITC treatments. *N* = 4–10 seedlings.

Glucosinolates are a group of secondary metabolites commonly found in members of the Brassicaceae family, including the cruciferous vegetables that have been part of the human diet for thousands of years (Halkier and Gershenzon, 2006). All known glucosinolate producing plants have at least one β-thioglucosidase often named myrosinase (Bones and Rossiter, 1996, 2006). Myrosinases hydrolyses glucosinolates into several potentially toxic compounds dependent on the reaction conditions and the presence of specifier proteins (Bones and Rossiter, 2006; Halkier and Gershenzon, 2006; Kissen and Bones, 2009; Kissen et al., 2009a; Wittstock and Burow, 2010; Kong et al., 2012). Glucosinolate degradation products are contributing to the distinct taste and flavor of cruciferous vegetables such as broccoli, mustard and wasabi and they constitute a potent defense system against herbivores and pathogens (Wittstock and Burow, 2010). To prevent constitutive production and potential damage to the plant cells, myrosinase is stored separately from its substrates in specialized cells called myrosin cells (Bones and Iversen, 1985; Bones and Rossiter, 1996; Halkier and Gershenzon, 2006; Kissen et al., 2009b; Wittstock and Burow, 2010). The hydrolysis products are produced upon attack by herbivores or pathogens when damage to the plant tissue and disruption of the cells causes myrosinase to come into contact with glucosinolates (Bones and Rossiter, 1996, 2006; Halkier and Gershenzon, 2006; Kissen et al., 2009b; Wittstock and Burow, 2010). The small sulfur-containing isothiocyanates (ITCs) are among the biodegradation products of glucosinolates. Due to their anticancer and chemopreventive properties, the ITCs have been the target of substantial research efforts over the last years (Cheung and Kong, 2010; Navarro et al., 2011). Little is known however of how ITCs affect plant cells or whether they have other physiological functions than as herbivore repellents or antimicrobials. Interestingly, several ITCs have been found to inhibit the growth of various plants species, including *Arabidopsis thaliana* (Wolf et al., 1984; Bialy et al., 1990; Yamane et al., 1992; Norsworthy and Meehan, 2005). Growth inhibition might partly be accounted for by ITC-induced disruption of the microtubule network, as shown in a recent study by our group to be inducible in *A. thaliana* by subjection to vapor phase of allyl ITC (AITC; Figure 1A) (Øverby et al., 2015a). Furthermore, AITC was found to induce stomatal closure in *A. thaliana* through a ROS dependent process (Islam et al., 2009; Khokon et al., 2011). Reduction of stomatal aperture is a much used defense strategy that prevents pathogen entry and water loss upon herbivore attack, possibly suggesting a function for AITC in this commonly employed defense pathway (Khokon et al., 2011). Further support for a role of AITC in ROS mediated processes comes from other recent results from our group showing rapid depletion of intracellular gluthathione (GSH) and activation of gluthathione S-transferase genes in *A. thaliana* after AITC exposure (Øverby et al., 2015b). Although numerous studies have shown ITCs to interfere with the cell cycle progression of cancer cells, no studies have to our knowledge targeted the effect of ITCs on the plant cell cycle. Despite research efforts over the last decades, our understanding of the progression and regulation of the plant cell cycle remains limited (Francis, 2011). The plant cell cycle machinery differs in certain aspects from that of other eukaryotes, yet the main drives of the cell cycle in plants, yeast and mammals are the same: the highly conserved cyclin dependent kinases (CDKs) (Stals and Inze, 2001; Inze and De Veylder, 2006). CDKs are activated by complex formation with cyclins, the levels of which fluctuates throughout the cell cycle by regulated transcription and proteolysis (Boudolf et al., 2006; Inze and De Veylder, 2006). CDKA promotes the G_1_/S transition by binding CycD and subsequently phosphorylate the retinoblastoma related (RBR) protein. This activates the RBR/E2F/DP pathway, leading to DNA replication and progression into the G_2_ phase of the cell cycle (Inze and De Veylder, 2006). Cyclin-dependent kinase inhibitors (CKIs) have been suggested as the main negative regulators of the G_2_/M transition in plants (Boudolf et al., 2006; Francis, 2011). *A. thaliana* encodes seven ICK/KRPs that can bind CDKs and cyclins, and the G_2_/M transition is likely to be driven by CDKB-induced release of CDKA from ICK2/KRP2 (Verkest et al., 2005; Boudolf et al., 2006; Inze and De Veylder, 2006). It is however, important to note that the possibility of CDKB directly driving the cell through G_2_/M has yet to be ruled out (Verkest et al., 2005). Completion of the G_2_ intermediate phase is followed by mitosis and cytokinesis in which the chromosomes are separated and the cell divides. Upon completion of cytokinesis, plant cells might progress to another round of DNA replication and cell division or enter the endocycle, an alternative cell cycle characterized by DNA replication without subsequent cell division (Figure 1B; Inze and De Veylder, 2006). Endoreduplication is common to many plant species and is associated with the onset of differentiation and cell expansion (Inze and De Veylder, 2006; Lammens et al., 2008). It has been suggested that inhibition of mitosis is sufficient for the cell to switch to the endocycle and correspondingly inhibitors of CDKs or cyclins such as the APC/C, WEE1, SIAMESE and ICKs/KRPs have been found to promote endocycle onset (Boudolf et al., 2004; Inze and De Veylder, 2006; Lammens et al., 2008). Interestingly, a recent study by Bao et al. linked cell cycle arrest to the onset of defense responses and expression of defense related genes (Bao et al., 2013). In the present study, we investigated the effect of non-lethal, growth inhibiting concentrations of vapor phase of AITC on the cell cycle of *A. thaliana*. We show that AITC induces a distinct cell cycle shift resulting in increased S-phase populations in *A. thaliana* seedlings. Furthermore, we show that AITC down-regulates the expression of mitotic genes, indicating a potential regulation of the plant cell cycle by a metabolite commonly regarded as a feeding deterrent.

## Results

### AITC inhibits growth of *A. thaliana* seedlings and causes disintegration microtubules

**Figure 2:**
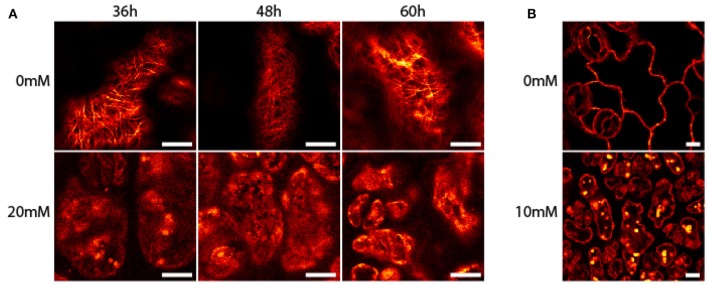
**Disintegration of***A. thaliana*** microtubules after treatment with AITC**. Transgenic seedlings were subjected to vapor of 0, 10, 20, or 30 mM AITC for 36, 48, and 60 h. The microtubule skeleton of pavement cells was analyzed by confocal microscopy. **(A)** Representative results of seedlings after 20 mM AITC treatment. **(B)** Cells subjected to 10 mM AITC for 60 h were smaller than mock-treated cells. Bar is 10 μM.

Seedlings of *A. thaliana* (6–7 day-old) were exposed to AITC at a developing stage where proliferation still is ongoing throughout the first two true leaves (Skirycz et al., 2011). As cells of *A. thaliana* can take up to 48 h to progress through the cell cycle, seedlings were exposed to AITC for 36, 48, and 60 h, thus ensuring enough time for all cells to go through the cell cycle at least once (Beemster et al., 2002). Exposure of 7 day-old seedlings of wild-type *A. thaliana* to vapor-phase AITC of 0, 10, 20, and 30 mM concentrations for 36, 48, and 60 h, revealed a dose-dependent growth inhibition consistent with previous reports (Figures 1C,D) (Hara et al., 2010; Øverby et al., 2015a). To study the severity of AITC-induced growth inhibition, seedlings were allowed to recover in an AITC free atmosphere for up to 11 days and seedling size was measured once a day during the first 4–6 days of recovery (Figures 1C,D). AITC treated seedlings remained smaller than control seedlings throughout the recovery period, with seedlings exposed to 20 or 30 mM AITC showing the most significant growth inhibition. Concentration of 1 M AITC has been shown to cause partial bleaching while 3.4 M AITC caused a substantial loss of chlorophyll and loss of green color (Øverby et al., 2015b). In the present study, seedlings that were subjected to vapor of 20 and 30 mM AITC for 36 h displayed only bleaching of cotyledons at day 2 after treatment was ended. Interestingly, seedlings that had been subjected to the same concentrations for 48 and 60 h displayed bleaching of cotyledons at the time when AITC exposure was stopped, although other chlorophyll containing organs remained green (Figure 1E). The microtubule cytoskeleton is important for cell division and expansion, both of which are required for plant growth and development (Liu et al., 2011). Exposure to vapor of 0.5 M and higher AITC concentrations has previously been shown to disintegrate microtubules in *A. thaliana* (Øverby et al., 2015a). We therefore hypothesized that disintegration of the microtubule network might contribute to the observed growth inhibition after exposure to vapor of millimolar concentrations of AITC. To that end, a transgenic *A. thaliana* line which expresses GFP-tagged α-tubulin was subjected to the same AITC treatments as above. Confocal microscopy of pavements cells of the first two true leaves revealed that all AITC treatments caused disintegration of the microtubule network (Figure 2A). The seedlings used in this study were subjected to AITC when 6 or 7 days-old, at which point cell division was still ongoing in most parts of the leaf. However, cells located at the tip of the leaf cease to divide and begin expanding in seedlings older than 8 days (Skirycz et al., 2011). Confocal microscopy showed that cells of AITC treated leaves were in average smaller than cells in non-treated leaves, indicating that AITC also prevented expansion of the cells (Figure 2B).

### AITC-induced cell cycle shift increases S-phase populations

**Figure 3:**
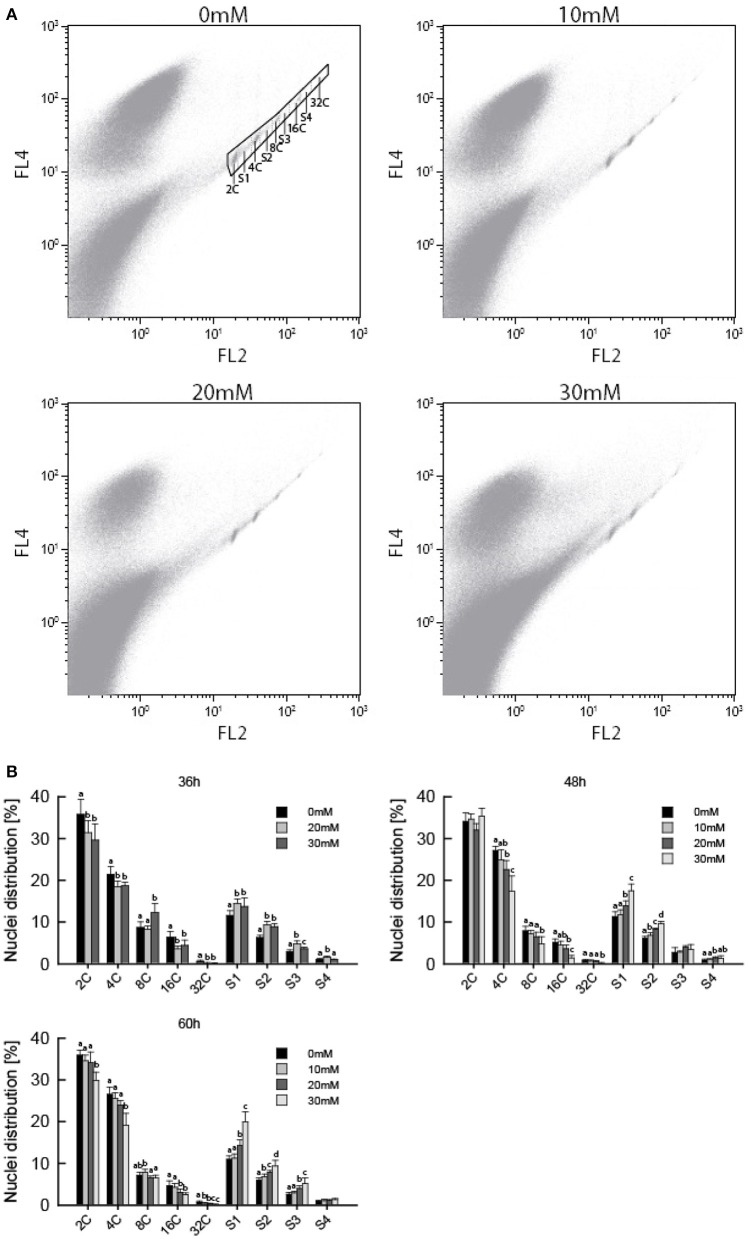
**Flow cytometric analysis of ***A. thaliana*** nuclei following AITC treatment**. Seedlings were subjected to vapor of 0, 10, 20, or 30 mM AITC for 36, 48, and 60 h before cell cycle distribution was analyzed by flow cytometry. **(A)** Biparametric dot plot of FL2 (575 nm) versus FL4 (695 nm) fluorescene emission with 488 nm exitation as detected by flow cytometry and extracted using the software Kaluza. The plots presented are representative plots from seedlings treated with 0, 10, 20, and 30 mM AITC for 48 h. An example of gating is shown in the 0 mM treatment plot. Gated signals comprised <1% of all signals detected. **(B)** Percentage of nuclei in each cell cycle phase for all treatments. Different letters indicates statistically significant differences between treatments within the same cell cycle phase, no letters indicates no significant difference. The means of 3 independent experiments with each 2–3 biological replicates are shown.

Several studies have shown ITCs to induce cell cycle arrest in cancer cells, which is often found upon treatment with microtubule inhibitors (Zhang et al., 2003; Navarro et al., 2011). We therefore hypothesized that AITC-induced disintegration of the microtubule network prevented the cells from undergoing mitosis, resulting in cell cycle arrest, reduced growth rate and smaller *A. thaliana* seedlings. Flow cytometry was used to assess the distribution of cells in the cell cycle. Due to the size of plant cells and the presence of a rigid cell wall, extracted nuclei were used instead of whole cells. This results in the loss of cells that have proceeded beyond prophase where the nuclear envelope breaks down, preventing the flow cytometry analysis from distinguishing the G_2_/M phase of the cell cycle. Furthermore, *A. thaliana* show endoreduplication, a process of repeated rounds of DNA replication that is not followed by cell division, resulting in nuclei with several copies of DNA. Therefore, the G_2_/M population is replaced with a population of cells containing 4 copies of DNA (4C). This population contains cells that will proceed to mitosis, remain at 4C or enter the endocycle. Mitotic arrest will increase the population of 4C cells if the cells are arrested prior to nuclear envelope breakdown. However, mitotic arrest after nuclear envelope breakdown will result in a reduction of the 4C population. Cells not in the S-phase of the cell cycle are from now on referred to as cells in the C-phase, with preceeding number referring to the number of DNA copies in the cell. To investigate the effect of AITC on the cell cycle distribution of *A. thaliana*, 6–7 day-old seedlings were subjected to vapor of 0, 20, and 30 mM AITC for 36 h and 0, 10, 20, and 30 mM AITC for 48 and 60 h, followed by extraction of nuclei and analysis by flow cytometry. The analysis revealed that *A. thaliana* cells ranges from 2C to 32C, consistent with previous reports (Figure 3A) (Galbraith et al., 1991; Galbraith, 2009). Interestingly, AITC induced a significant dose-dependent decrease in the 4C population resulting in a reduction from 21.5 to 18.5% after 36 h AITC treatment (20 and 30 mM AITC) and from 27.1% in the control to 24.8% (10 mM), 22.6% (20 mM), and 17.3% (30 mM) after 48 h of AITC treatment (Figure 3B). Similarly, a decrease from 26.5% in the control to 25.5% (10 mM), 23.9% (20 mM), and 19.2% (30 mM) was seen after 60 h of AITC treatment. Even though this suggests a mitotic cell cycle arrest after nuclear envelope breakdown, a similar decrease was found in all populations with different DNA copy numbers. Furthermore, a statistically significant dose-dependent increase of all S-phase populations was observed at all time points. The increase was most prominent in the S1 population, which in the case of the 36 h treatment increased from 11.4% in control seedlings to 14.5 and 13.7% for 20 and 30 mM AITC, respectively. After 48 h of AITC treatment the S1 population increased from 11.3% in the control to 11.7% (10 mM), 13.9% (20 mM), and 17.4% (30 mM). Similarly the S1 population increased from 11.0% in control seedlings to 11.3% (10 mM), 14.2% (20 mM), and 19.9% (30 mM) after 60 h of AITC treatment. Taken together, these results suggest that AITC, in a dose-dependent manner, induces a cell cycle shift toward larger S-phase populations and smaller non-replicating cell populations in *A. thaliana*. Despite being evident for all cell cycle phases, this trend was most prominent for the 2C, S1, 4C, and S2 phases which includes approximately 70% of the cells.

### Analysis of cell cycle related genes indicate inhibition of mitosis but not DNA synthesis

As shown by flow cytometry, AITC induced a cell cycle shift of increased S-phase populations while decreasing C-phase populations. To understand the role of cell cycle regulators in this shift we performed a transcriptional analysis of 16 genes encoding proteins involved in cell cycle regulation (Table 1). For this analysis 6–7 day-old *A. thaliana* seedlings were subjected to vapor of 20 and 30 mM AITC for 36, 48, and 60 h, which were the treatments that induced the most significant cell cycle shifts. CDKA is the only CDK found to be active in the G_1_ and S phases in plant cells and it promotes the G_1_/S transition by complex formation with CycD and subsequent activation of the RBR/E2F/DP pathway (Inze and De Veylder, 2006). Interestingly, *CDKA* expression was found to be up-regulated after exposure to vapor of 20 and 30 mM AITC for 36 and 48 h, but was down-regulated after 20 mM for 60 h (Table 2). Expression of other S-phase related genes included in this study (*CycD1;1, CycD3;1*, and *CycD4;1*) were too low to be reliably detected by our method. CDKA functions in coordination with CDKBs to promote the transition from G_2_ to mitosis, possibly by CDKB1;1 induced release of CDKA from ICK2/KRP2 inhibition (Verkest et al., 2005; Boudolf et al., 2006; Inze and De Veylder, 2006). Exposure to vapor of 20 mM AITC for 36 h and 30 mM AITC for 48 h resulted in down-regulation of *CDKB1;1* expression. Expression levels of *CDKB1;2* did not change, however *CDKB2;1* was down-regulated by both doses tested after 36 h of treatment and *CDKB2;2* was up-regulated after 48 h. Interestingly, none of the genes encoding mitotic inhibitors included in this study were up-regulated, including *ICK2/KRP2* (Table 2). However, expression of the mitotic cyclins *CycA1;1* and *CycA2;3* were down-regulated by 20 mM AITC for 60 h and 30 mM for 48 and 60 h. Furthermore, *CycA2;3* was down-regulated by 20 mM AITC for 36 h. Expression of the mitotic protein KNOLLE was also down-regulated, although not statistically significant for all treatments (Table 2). Taken together, these results indicate that AITC induces expression of S-phase related genes while down-regulating mitotic genes in *A. thaliana*.

**Table 1:** **Cell cycle regulators selected for transcriptional analysis**.

**Gene**	**Accession number**	**Function**
*CDKA*	At3g48750	Regulates the G_1_/S and G_2_/M transitions
*CDKB1;1*	At3g54180	Regulates the G_2_/M transition, ICK2/KRP2 inhibitor
*CDKB1;2*	At2g38620	Regulates the G_2_/M transition
*CDKB2;1*	At1g76540	Regulates the G_2_/M transition
*CDKB2;2*	At1g20930	Regulates the G_2_/M transition
*CycA1;1*	At1g44110	Active during G_1_/S and M, involved in DNA replication
*CycA2;3*	At1g15570	Active during G_1_/S and M, negatively regulates endocycle
*CycB1;1*	At4g37490	Mitotic cyclin regulating the G_2_/M transition
*CycD1;1*	At1g70210	Regulates the G_1_/S transition by binding to CDKA
*CycD3;1*	At4g34160	Regulates the G_1_/S transition. Interacts with CDKA and KRP1/ICK1
*CycD4;1*	At5g65420	Regulates the cell cycle at the G_1_/S and G_2_/M transition by binding to CDKA or CDKB
*DEL1*	At3g48160	E2F transcription factors inhibit expression of CCS52A2 during mitosis
*AtTCP15*	At1g69690	Transcription factor regulating expression of mitotic genes
*KNOLLE*	At1g08560	Involved in cell plate formation
*CCS52A2*	At4g11920	Subunit of APC/C, mitotic inhibitor
*Wee1*	At1g02970	Mitotic inhibitor activated by DNA damage
*ICK1/KRP1*	At2g23430	Inhibits mitosis by inactivating CDKs and cyclins
*ICK2/KRP2*	At3g50630	Inhibits mitosis by inactivating CDKs and cyclins
*SIAMESE*	At5g04470	Promotes entry into endocycle

**Table 2:** **Transcriptional analysis of cell cycle related genes of ***A. thaliana*** subjected to AITC treatment**.

**Gene**	**20 mM**						**30 mM**					
	**36 h**		**48 h**		**60 h**		**36 h**		**48 h**		**60 h**	
*CDKA*	4.3^*^	1.9–11.4	2.0^**^	0.4–4.2	0.3^*^	0.2–0.4	5.5^*^	2.2–14.4	3.7^*^	2.8–5.1	0.5	0.2–1.1
*CDKB1;1*	0.2^*^	0.1–0.5	0.6	0.3–1.0	0.7	0.5–1.0	0.8	0.4–2.3	0.1^**^	0.1–0.2	0.2^*^	0.1–0.3
*CDKB1;2*	1.4	0.8–2.5	0.8	0.5–1.4	0.5	0.3–0.8	1.7	0.9–2.9	1.8	1.2–2.8	0.7	0.5–1.0
*CDKB2;1*	0.1^**^	0.06–0.1	2.4	0.8–8.8	3.4	1.0–9.2	0.1^**^	0.09–0.2	1.0	0.3–3.2	1.5	0.6–3.9
*CDKB2;2*	0.7^*^	0.5–0.8	1.7^*^	1.1–2.3	1.0	0.8–1.3	0.8	0.6–1.1	1.8^*^	1.1–2.3	1.0	0.8–1.3
*CycA1;1*	1.1	0.7–1.8	0.5	0.2–1.1	0.6^*^	0.5–0.8	0.7	0.4–1.2	0.9^*^	0.8–1.0	0.3^*^	0.2–0.7
*CycA2;3*	0.6^*^	0.5–0.8	0.8	0.5–1.3	0.6^*^	0.5–0.6	0.5	0.2–0.9	0.3^*^	0.3–0.4	0.3^*^	0.2–0.5
*CycB1;1*	0.9	0.7–1.1	0.5	0.2–0.8	1.0	0.8–1.2	0.5	0.3–0.8	0.6	0.3–1.2	0.8	0.3–1.4
*DEL1*	1.2	0.8–1.8	0.7^*^	0.4–1.3	0.5^*^	0.4–0.5	0.1	0.7–1.7	1.1	0.9–1.3	0.5	0.3–0.8
*AtTCP15*	2.5	1.0–5.2	0.5	0.2–0.8	0.9	0.6–1.1	4.7	3.8–6.3	–	–	0.9	0.3–4.4
*KNOLLE*	0.6^*^	0.5–0.7	0.7^*^	0.6–0.9	0.8	0.5–1.1	0.6^**^	0.5–0.7	0.7	0.6–0.9	0.7^*^	0.6–0.8
*CCS52A2*	1.4	1.3–1.7	1.3	1.2–1.4	1.1	1.0–1.1	1.8	1.6–2.0	1.7	1.5–1.8	1.3	1.2–1.4
*Wee1*	0.2^*^	0.1–0.5	0.9	0.7–1.4	0.6	0.4–1.0	0.7	0.2–1.5	0.3^*^	0.2–0.4	0.2^*^	0.1–0.3
*ICK1/KRP1*	0.7	0.5–0.8	1.9	1.0–3.7	1.2	0.5–2.6	1.0	0.3–2.4	1.5	0.6–3.5	0.9	0.3–2.0
*ICK2/KRP2*	2.2	1.4–3.0	0.7	0.4–1.3	1.0	0.8–1.2	1.8	1.1–2.3	1.4	0.8–2.2	0.8	0.3–1.8
*SIAMESE*	1.2^*^	1.1–1.2	1.1^*^	1.0–1.2	0.9	0.9–1.0	1.0	0.9–1.1	1.1	1.0–1.2	1.0	0.9–1.0

Expression levels were determined by qPCR after subjection of A. thaliana seedlings to vapor of 0, 20, and 30 mM AITC for 36, 48, and 60 h. Values represent relative expression levels of the genes based on cycle threshold values obtained from 3–4 biological replicates using qPCR, followed by statistical analysis using the software REST. Asterisks indicates statistically significant difference from mock-treatment

* (P < 0.05,

** *P < 0.07)*.

### Analysis of DNA synthesis following AITC-exposure

**Figure 4:**
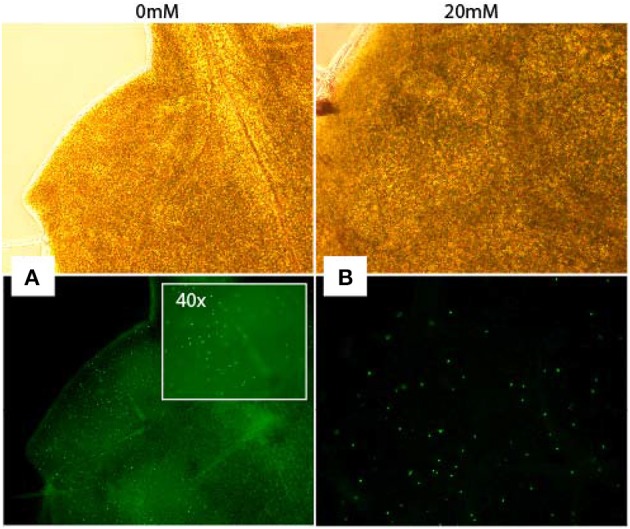
**Analysis of DNA synthesis in ***A. thaliana*****. Newly synthesized DNA of a mock-treated seedling **(A)** and a seedling subjected to 20 mM AITC for 36 h **(B)** visualized by EdU-staining of tissue for 4 h after treatments. Pictures were captured under light (top pictures) and fluorescence (bottom).

Increased S-phase populations could be the result of more cells undergoing DNA replication or be caused by increased initiation followed by failed completion of DNA replication in already existing cells. To study whether cells of AITC treated seedlings were able to replicate DNA, we employed a cell proliferation assay which allowed newly synthesized DNA to be visualized (Figure 4). Seedlings were subjected to vapor of 0, 10, 20, and 30 mM AITC for 36 and 48 h and incubated with the assay-component EdU for 1 h. Seedlings that had been exposed to AITC displayed no DNA synthesis, while newly synthesized DNA was visible in control leaves. However, after subjection to 10 and 20 mM AITC for 36 h followed by EdU staining for 4 h, newly synthesized DNA was found in both AITC treated and control leaves (Figure 4). Non-EdU treated seedlings were used as negative control and showed no new synthesized DNA regardless of incubation time. This indicated an AITC-induced reduction of DNA synthesis rate. Interestingly, after 4 h of EdU treatment, fewer of the leaves that had been exposed to vapor of 20 mM AITC for 36 h displayed DNA synthesizing cells than 10 mM treated- and control leaves. Taken together, these results suggest that AITC reduces the rate of DNA synthesis in a dose-dependent manner, although the interpretation of the assay was complicated by variation in the amounts of newly synthesized DNA in leaves from the same plants.

## Discussion

ITCs are known to function as herbivore and pathogen deterrents. However, recent studies suggest that these small metabolites have additional roles in plant defense (Khokon et al., 2011; Hara et al., 2013). In the present study, we report that non-lethal concentrations of AITC induced a shift in cell cycle distribution of *A. thaliana* leading to increased S-phase and decreased C-phase populations of cells. In a study by Hara et al. (2010) it was shown that concentrations of 10 and 100 mM AITC caused phytotoxicity in *A. thaliana* (Hara et al., 2010). However, in the present study vapor of 10 mM AITC for 36, 48, and 60 h did not cause any visible bleaching while 20 and 30 mM AITC caused bleaching of the cotyledons but not of the rosette leaves. These discrepancies are likely the result of different methods of AITC application. AITC is volatile and upon herbivore attack cells beyond the immediate wounding site are likely exposed to various concentrations of vapor phase AITC. Our application system models this form of AITC exposure, although all above ground organs were exposed to similar concentrations. In addition, the sustained exposure required to obtain any measurable effect on cell cycle in *A. thaliana* by AITC by our methodology is also likely to differ from the exposure in nature. The level of ITC exposure that plant cells encounter in nature is currently not known, although concentrations up to 100 mM has been suggested (Koroleva et al., 2000; Halkier and Gershenzon, 2006). However, as this estimate is based on the amount of glucosinolates contained in specialized cells it is unlikely that plant cells would be exposed to 100 mM ITCs (Hara et al., 2010). The physiological relevant concentrations of ITCs are likely to vary as different plant-herbivore or plant-pathogen interaction produces distinct collections of defensive metabolites that are often targeted specifically to the attacking herbivore or pathogen (Dixon, 2001; Bednarek and Osbourn, 2009; Bednarek et al., 2009; Clay et al., 2009). Determining what constitutes physiological relevant concentrations is further complicated by the spatial distribution of secondary metabolites involved in defense. Cells located close to the site of damage are likely exposed to other metabolites and higher concentrations than cells further away (Kliebenstein et al., 2005). Glucosinolate content in grounded tissue of *A. thaliana* has been estimated to 10 μmol/g fresh weight, and it has been suggested that this level may generate sufficient AITC to induce a stomatal closure in *A. thaliana* (Khokon et al., 2011; Hossain et al., 2013). Direct exposure of leaves from *A. thaliana* to 10–100 μM AITC induced a stomatal clure (Khokon et al., 2011). Although future studies should aim to estimate the exact AITC concentration in the gas-phase throughout the whole treatment using vapor exposure approach, theoretical calculation based on the vapor pressure of AITC (493 Pa) suggests an exposure of seedlings to around 2 μM AITC through gas-phase. In this study we exposed seedlings to low AITC concentrations that were non-lethal and only caused mild bleaching. Several studies have shown ITCs to cause cell cycle arrest of cancer cells, yet to our knowledge this has not previously been shown for plants. Our group has recently shown that AITC disintegrates microtubules in *A. thaliana* (Øverby et al., 2015a). To our surprise, despite inducing microtubule disintegration, AITC-exposure led to an increase of all S-phase populations suggesting an induction of DNA replication. Transcriptional analysis of genes encoding cell cycle regulators showed an up-regulation of *CDKA* by 20 and 30 mM AITC for 36 and 48 h, suggesting an initial response to AITC causing cells to enter the cell cycle. Furthermore, *CDKB1;1* was down-regulated after exposure to vapor of 20 mM AITC for 36 h and 30 mM for 48 and 60 h. Interestingly, genes encoding several mitotic proteins were down-regulated by AITC exposure, including genes encoding the mitotic cyclins CycA1;1 and CycA2;3 and the mitotic protein KNOLLE, which regulates cell plate formation during mitosis. The mitotic inhibitor ICK2/KRP2 has been suggested as an important regulator of the G_2_/M transition in plants. In this model ICK2/KRP2 binds and inhibits CDKA, thereby preventing progression into mitosis until CDKB1;1 induces the release of CDKA (Verkest et al., 2005; Boudolf et al., 2006; Inze and De Veylder, 2006). Interestingly, none of the mitotic inhibitors included in this study were up-regulated by AITC. In mammalian cells however, ITCs have been shown to bind to proteins through cysteine sulfhydryl groups and thereby regulate protein activity (Cheung and Kong, 2010; Zhang, 2012). Similar events are likely to occur in plants cells and further attempts to elucidate the effects of AITC on mitotic processes, should therefore target the level and activity of mitotic inhibitor proteins. The AITC-induced up-regulation of *CDKA* and down-regulation of *CDKB1;1* observed in the present study could suggest an induction of DNA synthesis followed by inhibition of mitotic entry. Interestingly, expression of transcripts encoding the mitotic inhibitor Wee1 was down-regulation by AITC treatment. As a part of the DNA damage check-point, Wee1 arrests the cell cycle by inhibition of CDKA and CDKBs when activated by DNA damage or certain stress conditions (Boudolf et al., 2006; Francis, 2007). The down-regulation of *Wee1* suggests that these responses are not involved in the observed AITC-induced cell cycle shift in *A. thaliana*. Interestingly, genes encoding several mitotic proteins were down-regulated after AITC exposure (Table 1). These results indicate that AITC inhibits mitotic processes, but this does not explain why cells accumulated in S-phases. Increased populations of cells in S-phases might be due to AITC induced DNA replication, but also to inhibition of DNA synthesis without affecting the onset of DNA replication. Although up-regulation of *CDKA* indicates AITC-induced entry into S-phase and the onset of DNA replication, the DNA synthesis visualization assay showed that AITC treated cells had reduced rate of DNA synthesis. Taken together, these results suggest that AITC exposure induces the cells to enter the cell cycle and start DNA replication. Simultaneously, the rate of DNA synthesis is reduced, causing increased S-phase populations and delayed seedling growth. Cell cycle arrest has previously been coupled to defense responses. In a study by Bao et al. it was shown that cell cycle arrest induced expression of defense associated genes (Bao et al., 2013). Furthermore, cell cycle arrest was found to increase resistance to pathogens, although the link between cell cycle arrest and defense responses is currently not understood (Bao et al., 2013). It has been suggested that the chromosomal rearrangements that takes place during DNA replication and mitosis could make certain defense genes more available to transcription, however, more research is needed to elucidate the role of cell cycle arrest in defense (Bao et al., 2013). Furthermore, it needs to be elucidated if changes in the cell cycle distribution induced by ITCs or other secondary metabolites indeed improve the defense activity of plants and whether these responses occur in nature. In conclusion, the present study shows that non-lethal concentrations of AITC induce a cell cycle shift in *A. thaliana*, causing cells to accumulate in S-phases. Previous reports show that the glucosinolate-myrosinase system has a role in plant defense against herbivores and pathogens (Bednarek and Osbourn, 2009; Bednarek et al., 2009; Clay et al., 2009), but a multifunctional role for the glucosinolate-myrosinase system has also been suggested (Bones et al., 1991). Our results add evidence to the growing data supporting a multifunctional role of glucosinolates and their degradation products in plants.

## Materials and methods

### Plant growth and AITC treatment

*A. thaliana* ecotype Columbia-0 (Col-0) wild-type and 35S::TUA-GFP (Ueda et al., 1999) seeds were disinfected by a chlorine/ethanol-based procedure and sowed on 9 cm petri-dishes with Murashige-Skoog (MS; Sigma, Norway) agar (MS, 2.15 g/l; sucrose, 20 g/l; agar, 6 g/l; pH 5.7). The seeds were vernalized in the dark for 4 days at 4°C and subsequently incubated at room temperature under a 16/8 h light/dark cycle for 6–7 days. For AITC (purity >95%; Sigma, Norway) treatment, a 9 cm petri-dish with 6–7 days old plants (lid removed) were placed in a 13 cm petri-dish containing a filter paper to which 200 μl AITC diluted in rape seed oil (from a local supermarket) was added. Rape seed oil without AITC was used as control. The petri-dish was sealed and the plants allowed to grow under the above-mentioned conditions for 36, 48, or 60 h. For analysis of the growth inhibitory effects of AITC the plant-containing petri-dish was removed from the larger petri-dish and plants allowed to recover under the above-mentioned growth conditions for 4–5 days. The seedlings were photographed and the software ImageJ was used to estimate seedling size.

### Confocal microscopy of microtubules

Six to Seven days old seedlings of *A. thaliana* Col-0 35S::TUA-GFP were subjected to vapor of 0, 10, 20, and 30 mM AITC for 36, 48, and 60 h. The first two true leaves were then immediately analyzed by confocal microscopy (Leica TCS SP5).

### Flow cytometry analysis

For flow cytometry analysis 6–7 days old seedlings of *A. thaliana* Col-0 wild-type treated with vapor of AITC for 36, 48, and 60 h were used. Immediately following exposure, roots were removed by scalpel and nuclei extracted from 20–70 mg of plant tissue by manual chopping with a razor blade for 2 min on a pre-cooled rack containing ice-cold Galbraith buffer (45 mM MgCl_2_, 30 mM sodium citrate, 20 mM MOPS, pH7 and 1% Triton X-100) using 1.5 ml buffer/100 mg plant tissue. The homogenate was filtered through a 30 μm nylon filter and subjected to RNase A (100 μg/ml; Sigma, Norway) for 30 min on ice. The homogenate was stained in the dark with propidium iodide (50 μg/ml; Sigma, Norway) for 30 min on ice. Samples were stored for up to 4 h and vortexed for 5 s prior to flow cytometry analysis. Care was taken to analyze the samples within 10 min of vortexing. The nuclei were analyzed with a Beckman-Coulter flow cytometer (Becton Dickinson LSR).

### qPCR

For analysis of the expression of genes encoding cell cycle regulators, 6–7 days old seedlings of *A. thaliana* Col-0 wild-type were subjected to vapor of AITC for 36, 48, and 60 h followed by harvesting tissue by removing roots and snap-freezing the remaining seedling in liquid nitrogen. RNA was extracted from ground tissue using Spectrum Plant Total RNA Kit (Sigma, Norway). RNase-Free DNase Set (QIAGEN, Norway) was used to prevent DNA contamination and RNA concentration was measured with NanoDrop 1000 (Thermo Scientific). QuantiTect Reverse Transcription Kit (QIAGEN, Norway) was used for cDNA synthesis and qPCR performed with SYBRgreen (Roche Applied Science, Norway) in a 96-well plate in Lightcycler 480 (Roche Applied Science) with the following program: preincubation step of 95°C for 5 min, followed by 45 amplification cycles (95°C, 10 s; 55°C, 10 s; 72°C, 10 s) and a melting curve analysis to check primer specificity. The housekeeping genes *clathrin* (At4g24550.1) and *TIP41-like* (At4g34270.1) were used for normalization. Lightcycler 480 Software (Roche) was used to calculate cycle threshold values and LinRegPCR and REST 2009 (QIAGEN) were used to calculate PCR efficiencies and relative expression values. Primer sequences are given in supplement (Table S1).

### EdU assay

For analysis of DNA synthesis, 6–7 day-old seedlings of *A. thaliana* Col-0 wild-type were subjected to vapor of AITC for 36 h prior to EdU incorporation using the Click-iT® EdU Alexa Fluor® 488 Imaging Kit (Invitrogen, Norway) according to the manufacturer's instructions. By subjecting seedlings to the thymine analog EdU, EdU is incorporated into the DNA of cells undergoing DNA replication. These cells can then be selectively visualized, thereby giving a snap-shot of active DNA replication. Seedlings were placed in eppendorf tubes with 500 μl 10 μM EdU and placed under the above-mentioned growth conditions for 1 and 4 h. After EdU incubation, the EdU solution was removed and seedlings washed twice with 1 ml PBS followed by fixation in 1 ml 3.7% formaldehyde in PBS for 1 h. Subsequently the fixative solution was removed and seedlings were washed twice with 1 ml 3% BSA in PBS followed by incubation in 1 ml 0.5% Triton X-100 in PBS for 20 min to increase the permeabilization of the cells. The permeabilization solution was removed and seedlings were washed twice with 1 ml 3% BSA in PBS before removal of the washing solution. The seedlings were then incubated for 30 min in the dark in 500 μl Click-iT reaction cocktail prepared according to the manufacturer's instructions. Following, the seedlings were washed once in 1 ml 3% BSA in PBS and the first two true leaves observed under a fluorescence microscope (Nikon Eclipse E800).

### Statistical analysis

The means of flow cytometry data for each cell cycle phase were compared using One-Way analysis of variance (ANOVA) with the Students-Newmans-Keuls *post-hoc* test. Equality of variance was tested with Lavene's test. If the assumption of equal variance was not fulfilled Kruskal-Wallis test with the appropriate *post-hoc* tests was used. All statistical analyses were done using MiniTab version 15 (www.minitab.com) and MedCalc for Windows version 12.4 (MedCalc Software, Mariakerke, Belgium).

### Conflict of interest statement

The authors declare that the research was conducted in the absence of any commercial or financial relationships that could be construed as a potential conflict of interest.
