# Supplementary material for: Allyl isothiocyanate affects the cell cycle of Arabidopsis thaliana
Source: Front Plant Sci. 2015 May 19;6:364. doi: 10.3389/fpls.2015.00364 (PMC4436579; doi:10.3389/fpls.2015.00364)
Supplement: Supplementary file 1 [file Table1.DOCX]

**Table S1 Primer sequences.**

| Gene | Forward primer sequence (5’-3’) | Reverse primer sequence (5’-3’) |
| --- | --- | --- |
| *CDKA* | TCCGTCGGTGTGCTAGTCTC | GTGCGGCGATCAATCAACAA |
| *CDKB1;1* | GTGTGCTTCACCGTGATCTT | TTGCTGCTCAGTTGGTGTTC |
| *CDKB1;2* | GTGTGCTTCACCGTGATCT | CCTCCTAATCATCTCGGCAAAG |
| *CDKB2;1* | GTCTCCATGAGGACGAAGAA | GGCACCAAGAAGAACCTCTG |
| *CDKB2;2* | GATTAGCCAGAGCCTTCACT | GGTTTCCATTGCGGGTATTC |
| *CycA1;1* | GCATCTTCGTGCTTCTGAGG | CAAGCCACACCAAGCAACTG |
| *CycA2;3* | AGAGCGGATTCAGGATTCAC | CAAAGCAGAGGCAAGGGAAC |
| *CycB1;1* | GGTGGAGTGGTTGATTGATG | TAGCCAGTGTGATGCTTGAG |
| *CycD1;1* | CGTCGCATGGATTCTCAAGG | GTAACTCGTTCGCTACACAG |
| *CycD3;1* | GAGAAATGGGACGATGAAGG | AGACGGTGGCATTTGTTGAG |
| *CycD4;1* | CTTCCTCCACGAGGGCATAC | CACAGCAGCAGCAACTTC |
| *DEL1* | AGACATCGTTTGCGTCAACC | GCCCAACCATTTCAATCCC |
| *AtTCP15* | CCTAAGAAGCCTCCTCCTAA | GGACTCTGACTCGTAGGTAA |
| *KNOLLE* | AGAACCGCTGTGACTAATGG | CCTGAATTGCTCGCGTGAGA |
| *CCS52A2* | GGCACTACGTGGAACACATT | TCCAACTCTTAGCCGATGAC |
| *Wee1* | GGACATTGGAGCGTCACTCT | AACTCTCCTGGCGACATCTT |
| *ICK1/KRP1* | GACTTCACGCACACGTAACC | TCCGATTCCGTTGGCATCTC |
| *ICK2/KRP2* | ACGACGACGGTGAAACGAAG | CCTCCACCAAGTGGCTCATC |
| *SIAMESE* | TCCGAGCCAACACCAACAGA | AGAGACGACGGTGTGGAAGG |
